# Management of Extramammary Paget's Disease: A Case Report and Review of the Literature

**DOI:** 10.1155/2013/436390

**Published:** 2013-11-14

**Authors:** Rosa Guerra, Subhasis Misra

**Affiliations:** ^1^Department of Surgery, School of Medicine at Amarillo, Texas Tech University Health Sciences Center, 1400 S Coulter Street, Amarillo, TX 79106, USA; ^2^Gastrointestinal and Hepato-Pancreato-Biliary Surgery, Division of Surgical Oncology, Texas Tech University Health Sciences Center, 1400 S Coulter Street, Amarillo, TX 79106, USA

## Abstract

Extramammary Paget's Disease (EMPD) is a rare condition of the skin that often involves the vulva, perianal region, scrotum, penis, and axilla. Although prognosis is generally favorable, it can be associated with neoplasms of the bladder, urethra, prostate, and rectum. This report presents a case of scrotal EMPD that failed treatment with imiquimod 5% cream and discusses benefits and complications of available treatment options. The variation of treatment success emphasizes the importance of further research.

## 1. Introduction

Extramammary Paget's Disease (EMPD) is a rare neoplastic condition of the skin or its underlying appendages commonly found in the vulva, perianal region, scrotum, penis, and axilla [1]. Clinically the condition presents as a well-demarcated, thickened, pruritic, erythematous, or white scaly plaque with irregular borders [2, 3]. Microscopically EMPD involves large cells with vacuolated cytoplasm and centrally located nuclei characterized as Paget cells [4]. The disease is categorized into primary or secondary EMPD with primary EMPD originating from intraepidermal cells and secondary EMPD coming from an underlying neoplasm [1, 4]. Neoplasms of the bladder, urethra, and prostate are associated with EMPD involving the external genitalia, while rectal adenocarcinoma is associated with perianal EMPD [5, 6].

The true incidence is still unclear but Karam and Dorigo found the median age at diagnosis to be 72 years with EMPD predominately occurring in Caucasians and women having a higher occurrence than men. Prognosis is generally favorable; however, older age, advance stage, and treatment modality can be associated with worse outcomes [1]. Although surgery is not always a feasible option for patients, surgical resection with clear margins is considered to be the standard of care. Other treatment options for EMPD involve, imiquimod 5% topical cream, modified peripheral Mohs surgery, and radiation therapy [1–3, 7, 8]. Many case studies have reported successful outcomes with 5% imiquimod cream in patients who did not undergo surgical treatment. In this paper, we present a case of scrotal EMPD that failed treatment with imiquimod 5% cream and discuss the benefits and complications of other treatment plans.

## 2. Case Report

**Figure 1:**
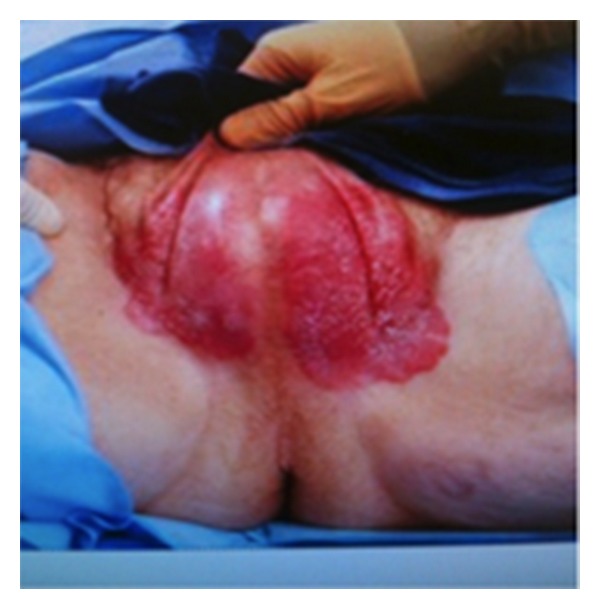
EMPD involving the scrotum and left thigh.

A 73-year-old white male presented with a 2-year history of a pruritic, erythematous lesion over his scrotum. Physical examination revealed an erythematous plaque extending over the right and left scrotum with an uninvolved, 1.5 cm strip at the median raphe for a distance of 7 mm on either side. The involved area contained whitish superficial exudates but did not involve the base of the penis nor extend anywhere near the anoderm. The left side of the plaque extended 2 cm onto the left thigh; however, no involvement of the right thigh was noted (Figure 1). No inguinal lymph nodes were palpated on physical examination and the testes were descended bilaterally.

His past medical history was significant for a right lower extremity deep venous thrombosis with pulmonary embolism one year prior to presentation for which he is taking Coumadin. He is also taking Albuterol and Symbicort for reactive airway disease. His past surgical history involved a vasectomy and epigastric hernia repair eleven years prior to presentation. His family history was negative for melanoma, colorectal cancer, or prostate cancer; however, his mother was diagnosed with breast cancer at age 60.

The patient was advised to undergo a screening colonoscopy and diagnostic cystoscopy due to the associated complication of EMPD with colon cancer and cancer of the bladder. After both procedures revealed unremarkable results the patient underwent mapping biopsies and the specimens were sent to pathology for analysis.

## 3. Pathology Report

**Figure 2:**
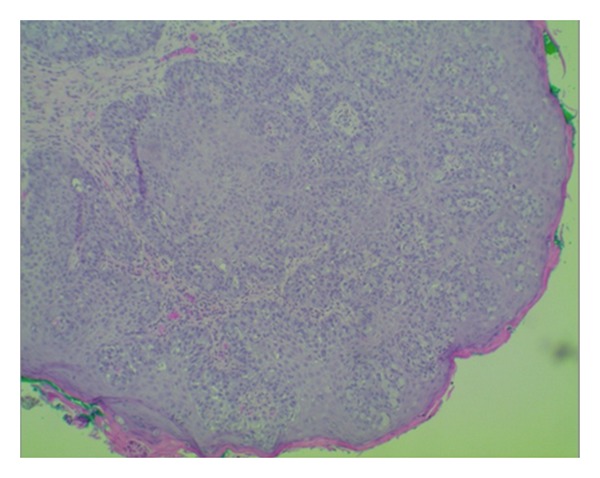
Histological image (low power) of epidermis infiltrated by suprabasal small nests and single epithelial cells with abundant vacuolated cytoplasm highlighted by mucicarmine special stain.

**Figure 3:**
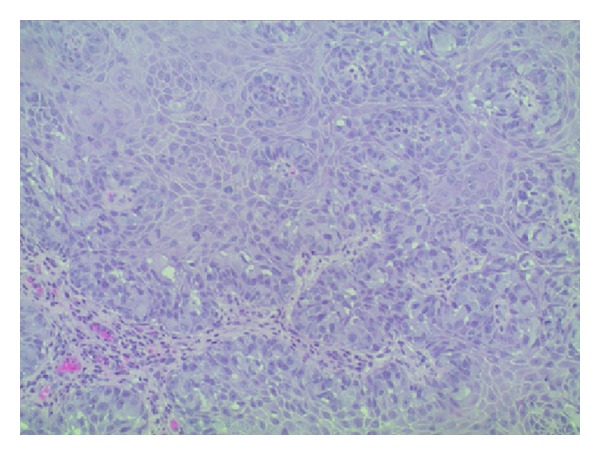
Histological image (high power) of epidermis infiltrated by suprabasal small nests and single epithelial cells with abundant vacuolated cytoplasm highlighted by mucicarmine special stain.

Histological examination showed fragments of skin with focal ulcerations and parakeratosis. The epidermis was infiltrated by suprabasal small nests and single epithelial cells with abundant vacuolated cytoplasm highlighted by mucicarmine special stain which is characteristic of Extramammary Paget's disease (Figures 2 and 3). There was no evidence of underlying malignancy noted; however, the dermis showed both mild acute and chronic focal inflammations.

## 4. Treatment Plan

**Figure 4:**
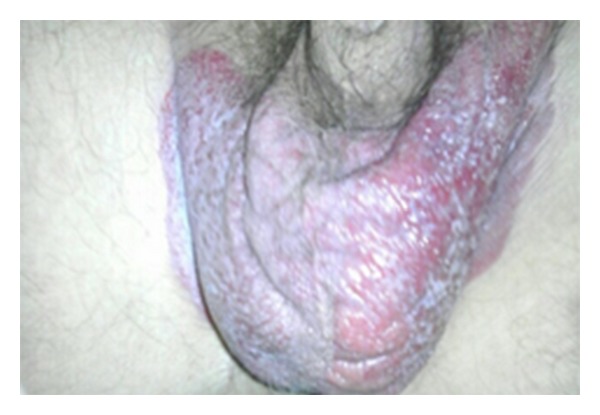
EMPD involving the scrotum and left thigh after two rounds of imiquimod 5% topical cream.

It was explained to the patient that surgical resection is the standard of care for EMPD. Due to personal preferences, the patient elected for medical management. Imiquimod 5% topical cream was applied three times a week for 16 weeks. After 16 weeks of the treatment, moderate improvement was noted. One-month after treatment, physical examination revealed the erythematous plaque of the left hemiscrotum showing moist desquamation with white exudates and the right hemiscrotum showing a dry, erythematous plaque with apparent epithelialization. Involvement of the median raphe was still not observed, but the plaque extended onto the skin of the pubis and was sharply demarcated beneath the pubic hair. A second round of imiquimod 5% therapy was initiated but was subsequently stopped four weeks into the treatment due to medication side effects of irritation and burning. After four months without any treatment, the topical imiquimod 5% cream was restarted. Currently, 22 weeks of treatment have passed and minimal improvement was noted on his last physical examination (Figure 4). The patient is in the process of weighing the risks and benefits of other treatment options but still declines to have surgery.

## 5. Discussion

Although the exact mechanism of action of imiquimod is unknown, it is used as an immune response modifier to stimulate innate and acquired immunity, in addition to Langerhan cell migration to regional lymph nodes by enhancing antigen presentation to naive T-lymphocytes [3, 9]. Currently, it is FDA approved for topical treatment of dermatological pathologies such as actinic keratosis, superficial basal cell carcinoma, and genital warts. Several case reports however have demonstrated its success in the treatment of EMPD [3, 10–12]. The treatment typically involves application of imiquimod 5% cream three times a week for a range of 6–16 weeks in order to avoid adverse effects. Side effects reported include local irritation, superficial erosion, ulcerations, pruritis, and pain in addition to generalized flu-like symptoms, fatigue, myalgia, and headaches.

Unfortunately, even after two rounds of treatment our patient did not show significant signs of improvement from topical application of imiquimod 5% cream. He had to interrupt the second round of treatment because the side effects involving irritation and burning were too debilitating. Failure with imiquimod treatment was reported in very few cases, and the contributing factors to unsuccessful treatment was not fully understood [7]. It was postulated that failure could have been attributed to the presence of invasive disease missed on initial or subsequent biopsy and complicated by lesion thickness variability. Our patient reported having difficulty treating the left hemiscrotum because the imiquimod cream did not apply well to his moist skin. Failure of proper adherence to the lesion could have attributed to inadequate penetration of the topical cream.

Surgical resection is the standard of care for EMPD and if selected would likely involve wide local resection and reconstruction via various modalities such as a scrotal skin flap or a gracilis muscle flap [13, 14]. Although Lee and Qin et al. reported success with surgical resection, the rate of recurrence after surgery is high and can range from 20 to 60% because the disease often extends beyond the visible borders of the tumor [2]. Using a Mohs technique to evaluate specimen margins has reduced the rates of reoccurrence to approximately 27% when compared to wide local surgical excision [15]. Mohs microsurgery can be time consuming when large EMPD lesions are involved, so a modified peripheral Mohs technique has been applied where only the peripheral specimen was histologically evaluated and the central specimen was removed without analysis. Unfortunately, this modified technique assumes the lesion consists of a uniform depth and incorrect assumptions can be critical to prognosis [2]. If the use of the Mohs technique is not available during surgical resection, markers for Ki67 and periodic acid-schiff (PAS) can be used to evaluate specimen margins. Feng et al. concluded in their study of 64 patients that the positive coexpression of Ki67 and PAS required wider excision (3 cm from the border of the lesion) than standard recommendations in order to ensure margins were negative and thus reducing the incidence of reoccurrence [4].

Radiation therapy could also be offered as an alternative treatment option, although reports the effectiveness of radiation therapy has shown variable outcomes. One case involving EMPD of the scrotum reported successful treatment with radiation therapy over a 3 month period after failure of local excision and imiquimod therapy [8]. Contrary to the aforementioned case report, a cohort analysis of 1439 patients found radiation therapy to be less effective. The mean disease-specific survival (DSS) time for patients receiving radiation (DSS of approximately 11 years) was significantly shorter when compared to patients who did not receive any radiation therapy (DSS of approximately 28 years) [1]. Interpretation of this cohort analysis is difficult because radiation fields, involvement of draining lymph nodes, doses, schedules, and sources were not standardized between patients. The study was also based on patients with invasive EMPD and the effect of radiation therapy for EMPD confined to the epidermis is not clearly known. Similar to radiation treatment, photodynamic therapy could provide patients with another less invasive treatment option, but further investigation needs to be explored [16].

## 6. Conclusion

In conclusion, EMPD is a rare disease involving the skin and apocrine glands of the vulva, anoderm, scrotum, penis, and axilla. The rarity of EMPD makes research for available treatment options difficult. Currently, the standard of care involves surgical resection; however, there is a high rate of reoccurrence and patients are not always medically or psychologically capable of tolerating surgery. The use of Mohs microsurgery or the modified peripheral Mohs technique have helped reduced the rated of reoccurrence. Other treatment options include imiquimod 5% cream, radiation therapy, and phototherapy, although successfulness of results often vary and warrant further research.
